# Assessment of the developmental success of *Anopheles coluzzii* larvae under different nutrient regimes: effects of diet quality, food amount and larval density

**DOI:** 10.1186/s12936-018-2530-z

**Published:** 2018-10-22

**Authors:** Patric Stephane Epopa, Hamidou Maiga, Domonbabele François de Sales Hien, Roch Kounbobr Dabire, Rosemary Susan Lees, Jeremie Giles, Frederic Tripet, Thierry Baldet, David Damiens, Abdoulaye Diabate

**Affiliations:** 1 Institut de Recherche en Sciences de la Santé/Centre Muraz, Bobo-Dioulasso, Burkina Faso; 2 Liverpool School of Tropical Medicine (LSTM), Liverpool, UK; 3 Insect Pest Control Laboratory (IPCL) of the FAO/IAEA Joint Division, Vienna, Austria; 4 Centre for Applied Entomology and Parasitology, School of Life Sciences, Keele University, Staffordshire, UK; 5 ASTRE, CIRAD, INRA, Univ Montpellier, Montpellier, France; 6 Institute of Research for Development, Montpellier, France

**Keywords:** Diets, Larvae density, Pre-imaginal developmental time, Wing length, Emergence rate, Mass-rearing, *Anopheles coluzzii*

## Abstract

**Background:**

In a context of increasing resistance of both vectors toward main classes of insecticides used in public health and parasites toward anti-malarial drugs, development of new and complementary molecules or control approaches is fundamental to achieve the objective of controlling or even eliminating malaria. Concerning vector control, the sterile insect technique and other genetic control approaches are among promising complementary tools in an integrated management strategy for malaria control. These approaches rely not only on a good understanding of vector biology (especially during larval stages), but also on the availability of adequate supplies and protocols for efficient mosquito rearing. The aim of this study was to assess the factors impacting the life history of *Anopheles coluzzii* mosquitoes at the larval stage, in the context of genetic and sterile insect approaches to control malaria vectors.

**Methods:**

The effect of different larval diets and larval rearing densities on the development of *An. coluzzii* were evaluated in the laboratory. Emergence rate (ER), pre-imaginal developmental time (DT) and adult wing length (WL) were measured under different food regimes. Four diets were tested among which three were provided by the Insect Pest Control Laboratory (IPCL) of the FAO/IAEA Joint division.

**Results:**

Data showed significant differences in the quality of the different diets and suggested a negative density dependence in all three life history parameters measured under tested rearing conditions. ER and WL increased with food availability, but decreased with increasing larval density. Conversely DT was shortened with increasing food availability but increased with larval density. These data demonstrates intraspecific larval competition modulated by food amount and space availability. Of the four diets tested, the one made of a mix of tuna meal, bovine liver powder, brewer’s yeast, squid liver powder and vitamin mix (diet 2) yielded the best results as it produced a good balance between ER, DT and WL. Food availability for optimal development (highest survival at shortest time) was in the range of 180–400 µg/larvae/day for the three diets provided by the IPCL.

**Conclusion:**

There is an interaction between diet type, diet concentration and larval density. Best results in terms of optimal larvae development parameters happen when moderately high values of those three variables are observed.

## Background

In spite of significant efforts invested in control (vector control, disease treatment, chemoprevention, health system improvement, etc.), malaria is still an important public health issue worldwide, but particularly in Africa. Recent data show that about 216 million malaria cases and 445,000 deaths due to malaria were registered worldwide during the year 2016, most of which (about 91%) occurred in sub-Saharan Africa [1]. Malaria control programmes in Africa rely heavily on the use of pyrethroids and carbamates in insecticide-treated nets (ITNs) and indoor residual spraying (IRS) [2]. Resistance to these insecticide classes has emerged in anopheline mosquitoes [1, 3] and its rapid spread suggests that it will become a major obstacle to vector control [2, 3]. Alternatives to insecticides are being explored, including biological control methods, such as the use of natural enemies [4, 5], genetic control methods such as the release of insects carrying cytoplasmic incompatibility [6, 7], homing endonuclease genes [8–11] and the conventional sterile insect technique (SIT) [12, 13].

The great diversity of the anopheline species involved in malaria transmission renders vector control more difficult and so for effective control, a good understanding of the bio-ecology of all the different vector species is required, particularly at their larval stage. In fact, the larval stage is the only stage during which density-dependent competition occurs [14–17]. Most of vector control methods directly or indirectly affect larval density in the natural habitat of the target species but do not totally eradicate the larvae, especially in the early phases of implementation. Reduction of larval density is likely to modify natural competition dynamics and alter larval survival and development rate, which may affect adult density and, therefore, disease transmission.

Several studies have been carried out to characterize the bio-ecology of anopheline species, and the consequences of varying larval density and resource availability on larval survival [17–21]. These studies provided important information on larvae dynamics. Nevertheless, little information is available about the effects of varying larval diet type on *Anopheles coluzzii* larval biology, and further studies could provide important information to improve the effectiveness of larval control. A good understanding of larval biology is also useful to those seeking to develop and implement better larval culture methods for the production of large numbers of mosquitoes that might be needed for genetic control methods such as the SIT.

The Insect Pest Control Laboratory (IPCL) of the FAO/IAEA Joint division is involved in a number of insect pest control programmes, using the SIT [13] and has developed different diets that may improve larval mass production in the laboratory. The current study is reporting on the effect of two of these diets on the survival rate of *An. coluzzii* larvae. Specifically, diet type, diet concentration, larval density and the interaction between these three parameters were assessed for their impact on survival rate, developmental time and adult body size.

## Methods

This study was carried out during the period of January to May 2012, at the medical entomology research unit of IRSS (Institut de Recherche en Sciences de la Santé, Bobo-Dioulasso, Burkina Faso). Diet preparation, wing dissection and measurement were done in the laboratory. Mosquito rearing and measurement of larval life history parameters were done inside the insectary.

### Diet preparation

Four different diets were tested in this study. Diet 1 was made of tuna meal (“TM”, supplied by T.C. Union Agrotech co ltd, Bangkok, Tailand), bovine liver powder (“BLP”, supplied by MP Biomedical, Solon, OH, USA) and vitamin mix (“VM” which was the Vanderzant vitamin supplied by BioServ, Frenchtown, IL, USA). Diet 2 had the same ingredients to which were added brewer’s yeast (“BY”, supplied by MP Biomedical, Solon, OH, USA) and squid liver powder (‘SLP’, supplied by T.C. Union Agrotech co ltd, Bukkalo, Tailand). These two diets were developed and provided by the IPCL in January 2012 [22, 23]. Diet 3 or “Koi” (formally used in routine for mosquito mass rearing in the IPCL and provided by this institution) was the fish food Koi Floating Blend^®^ (Aquaricare^®^, Victor, New York, USA, no longer available) [23], and diet 4 or “Tetramin” (used here as a control) was the fish food Tetramin^®^baby, used for routine larval rearing in IRSS laboratory.

The diets’ ingredients were grounded sufficiently to pass through a 224 µm standard sieve, using a planetary ball mill PM100 (Retsch^®^ GMBM, Haan, Germany). Final particle size were between 50 and 150 µm. Ingredients’ powder had been mixed to constitute the corresponding diets (Table 1). Four concentrations (0.5, 1, 2 and 3% (w/v)) of each diet were prepared using the appropriate weight of dry powder with deionized water. The suspensions were then homogenized for 10 min every hour for 7 h, using a stirrer. The suspensions were stored as aliquots of 1500 µl at − 20 °C until required.

**Table 1:** Ingredient proportions (%) in each diet

Ingredient/diet	Diet 1	Diet 2	Diet 3	Diet 4
Tuna meal (TM) (%)	50	25	0	0
Bovine liver powder (BLP) (%)	50	25	0	0
Squid liver powder (SLP) (%)	0	25	0	0
Brewer’s yeast (BY) (%)	0	25	0	0
Vitamin mix (VM) (additive)	4.6 g/l	4.6 g/l	0	0
Koi Floating Blend^®^	0	0	100	0
Tetramin^®^baby	0	0	0	100
Total (%)	100	100	100	100

### Maintenance of laboratory colony

The strain of *An. coluzzii* used in all experiments was colonized in August 2008 from gravid female adults collected in village 7 of the Kou valley (VK7) in western Burkina Faso. The colony has been maintained since that time in the insectary of the IRSS in Bobo-Dioulasso, Burkina Faso.

For general stock-keeping purposes, this strain was reared in a climate-controlled room at a temperature fixed at 27.4 °C (± 0.2) and a relative humidity of 76.3% (± 3.2) (95% CIs of the mean). The light regime was LD 12/12 h photoperiod, including dusk (1 h) and dawn (1 h). Larvae were reared in plastic trays (about 30 cm diameter) and fed with the fish food Tetramin^®^baby. Pupae were collected and placed in small plastic cups inside a fresh adult cage to emerge. Adults were kept in 30 × 30 × 30 cm insect cages (produced locally) and continuously supplied with 5% (w/v) glucose solution (made with deionized water). Females were blood fed on a live rabbit once weekly. Gravid females were allowed to oviposit in plastic Petri dishes containing a wet sponge covered with filter paper. Eggs were collected and hatched in plastic trays (about 30 cm diameter) containing spring water (1 l per tray).

### Experimental procedure

**Fig. 1:**
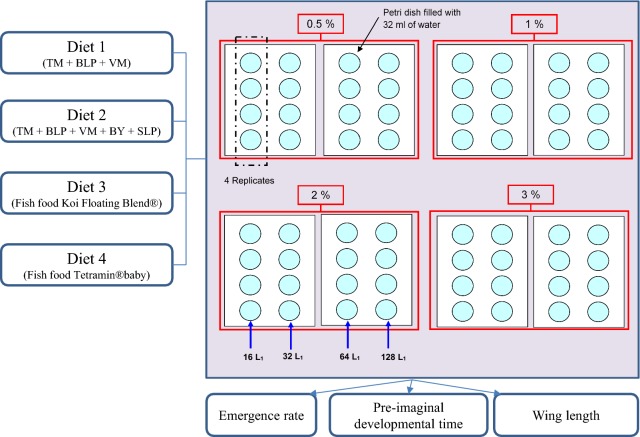
Experimental design. 4 different types of diets were assessed at the same time for their effects on three parameters: pre-imaginal developmental time (time needed to reach adulthood), emergence rate (number of adults that successfully emerged from an initial number of first instar larvae), and wing length as a proxy measure of adult body size. For each diet, 4 different concentrations (0.5%, 1%, 2% and 2%) were tested, each on 4 larval densities (16, 32, 64 and 128 first instar larvae) by adding 0.64 ml of corresponding diet per day. 4 replicates were used for each larval density. *TM* tuna meal, *BLP* bovine liver powder, *VM* vitamin mix, *BY* brewer’s yeast and *SLP* squid liver powder. Diet concentrations are expressed in percentage of weight (g) per volume (l)

In this experiment, 4 types of diets (diet 1, diet 2, Koi and Tetramin), 4 amounts of food (0.32, 0.64, 1.28 or 1.92 mg diet per day corresponding to a diet concentration of 0.5%, 1%, 2% and 3% w/v), and 4 larval densities (16, 32, 64 and 128 first instar larvae in 32 ml) were tested, a total of 64 treatments (4 diets × 4 concentrations × 4 densities) (Fig. 1). For each treatment 4 replicates were performed. All experiments were conducted in laboratory conditions as described above, following the protocol of Gilles et al. [21].

Eggs from *An. coluzzii* strain were collected on day 1 of the experiment and hatched in deionized water. Date and time of hatching were recorded. Four hours after hatching, 16, 32, 64 and 128 first instar larvae (L1) were hand-picked into a 50 ml graduated beaker using a plastic pipette. The beaker was filled with deionized water to the 32 ml mark and then poured into a clean brand new 9 cm diameter Petri dish, appropriately labelled (resulting on a water depth of 0.5 cm). The Petri dishes were set on a rack and larvae fed according to their designated treatment (Fig. 1). The Petri dishes remained covered with the lids supplied all along the experiment and their positions were changed every day to compensate for any localized differences that may exist on the rack (temperature gradients, for example). Diet (0.32, 0.64, 1.28 or 1.92 mg diet per day) was added to each Petri dish according to treatment by adding 640 µl of a 0.5, 1, 2 or 3% (w/v) slurry of diet in water.

Petri dishes were inspected for pupation in the morning (8 a.m.), at noon and in the afternoon (4 pm) and the pupae collected from each Petri dish were placed in a plastic cup covered with a net and left to emerge. The number of pupae, date and time of pupation, and the number and sex of adults that emerged were recorded for each Petri dish until no live larvae remained. Just after emergence, mosquitoes were put into cryotubes, labelled and stored at − 80 °C for wing dissection.

The effects of diet composition, diet concentration and larval density on larval development were studied. Specifically, adult emergence rate (ER), pre-imaginal developmental time (DT) and wing length (WL) of newly emerged mosquitoes were recorded. The emergence rate from each Petri dish was defined as the ratio of adults that successfully emerged to the number of first instar larvae initially introduced into the Petri dish. The pre-imaginal developmental time was defined as the time between egg hatching (L1) and the emergence of adults. For wing length, the mean length of the right and left wings was recorded. Each wing length was measured as the distance between the alula basis and the distal extremity of the 2.2 radial wing branch [24, 25] using Image software [26].

### Statistical analysis

The suitability of the different diets was judged by the emergence rate (number of adults that successfully emerged from an initial number of larvae), the pre-imaginal developmental time (time needed to reach adulthood) and wing length as a proxy measure of adult body size. Logistic regression analysis was performed to test the effects of diet type (diet 1, diet 2, Koi and Tetramin), diet concentration [C1 = 0.5%, C2 = 1%, C3 = 2% and C4 = 3% (w/v)] and larval density (D1 = 16, D2 = 32, D3 = 64 and D4 = 128 first instar larvae) taken separately and their interactions on the probability of adult emergence. Pre-imaginal developmental time and wing length were found not to follow a normal distribution (Kolmogorov–Smirnov test, *P* < 0.05) and their variances were not homogeneous (Fligner-Killeen test, *P* < 0.05), hence a logarithmic transformation was done to proceed with an ANOVA test. ANOVA was used for emergence rate, pre-imaginal developmental time and wing length to assed the influence of main variables (diet type, diet concentration and larval density) and their interaction, using deletion testing from an initial maximal model. Data were entered into Excel (Microsoft, 2007) and statistical analysis performed using R 3.3.1 software [27].

## Results

### Pre-imaginal developmental time

**Fig. 2:**
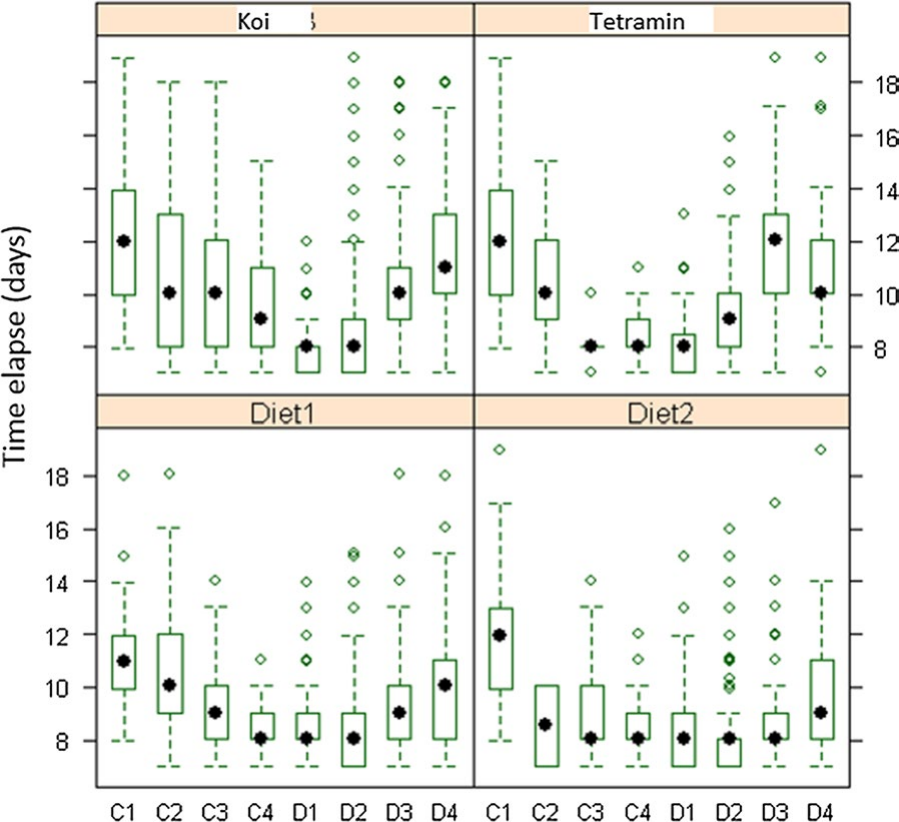
Impact of diet concentration and larval density on larval developmental time (DT) for each type of diet. The figure plots the combined effects of diet type, diet concentration (amount of food provided) and larval density on pre-imaginal developmental time (time needed for a young first instar larva to reach adulthood). The box extends between the 25th and the 75th percentile (across the inter quartile range) and the mean is denoted by a dot

Overall the pre-imaginal developmental time was estimated at 9.65 days and no significant difference was found between males and females (t = 0.05; *df* = 1; *P* = 0.0955). Significant effects of diet, diet concentration and larval density as well as of their interactions on pre-imaginal developmental time were observed (Table 2). A two by two comparison of the mean pre-imaginal developmental time between the four diets is shown in Table 3. Diet 2 produced the shortest pre-imaginal developmental time (8.89 days) followed by diet 1 (9.18 days) and Tetramin (10.19 days); larvae fed with Koi took the longest to develop to emergence (10.31 days). The specific effect of type of diet, accounting for diet concentration and larval density, on the pre-imaginal developmental time is shown in Fig. 2. Overall the pre-imaginal developmental time was longer at the lowest diet concentrations for all the diets and this time became shorter with increased food availability. Inversely, high larval density increased pre-imaginal development time.

**Table 2:** Effect of diet type, diet concentration, larval density and their interactions on the pre-imaginal developmental time

Source	*Df*	*F*	*P*
Diet	3	283.4	< 0.0001**
Concentration	3	411.2	< 0.0001**
Density	3	840.7	< 0.0001**
Diet–concentration	8	9.4	< 0.0001**
Diet–density	9	51.9	< 0.0001**
Concentration–density	9	33.9	< 0.0001**
Diet–concentration–density	24	4.4	< 0.0001**

The table describe the results of the logistic analysis (ANOVA) for the effect of diet type (“Diet”), diet concentration (“Concentration”) and larval density (“Density”) on the pre-imaginal developmental time of *An. coluzzii* mosquitoes. *Df* is the degree of freedom, *F* represents the value of the statistical test and *P* is the P-value of the test. The symbol “–” indicates the interaction between indicated sources. The symbol “**” indicates a significant effect

**Table 3:** Two by two comparison of the effect of diet type on pré-imaginal developmental time and wing length

Parameter	Sources compared	*Diff.*	*Lwr*	*Upr*	*P adj.*
Effect on pre-imaginal developmental time					
	Diet 2–diet 1	− 0.031	− 0.052	− 0.009	0.001**
	Diet 3–diet 1	0.108	0.089	0.127	0.000**
	Diet 4–diet 1	0.099	0.073	0.125	0.000**
	Diet 3–diet 2	0.138	0.118	0.159	0.000**
	Diet 4–diet 2	0.129	0.103	0.157	0.000**
	Diet 4–diet 3	− 0.009	− 0.034	0.016	0.811
Effect on wing length					
	Diet 2–diet 1	0.010	− 0.008	0.028	0.439
	Diet 3–diet 1	− 0.025	− 0.042	− 0.008	0.001**
	Diet 4–diet 1	− 0.023	− 0.046	0.001	0.061
	Diet 3–diet 2	− 0.035	− 0.053	− 0.017	0.000**
	Diet 4–diet 2	− 0.033	− 0.058	− 0.009	0.002**
	Diet 4–diet 3	0.002	− 0.022	0.026	0.997

The table describe the results of the Tukey’s honest analysis for comparison of the difference in the mean value of pré-imaginal developmental time (above) and wing size (bellow) between the assessed diets

*Diff.* mean differences, *Lwr* lower limit of the confidence interval, *Upr* upper limit of the confidence interval, *P adj.* adjusted value of P

The symbol “**” indicates a significant difference between the mean values (*P adj.* ˂ 0.5)

### Emergence rate

In the experiment male and female datasets were pooled for no significant difference has been observed between male and female results (*df* = 1, *P* = 0.215, χ^2^). Emergence rates were significantly different between diets, diet concentrations and larval densities. Significant two and three way interactions were seen between the three variables except for the interaction between diet and larval density (Table 4). For some reason which could not be elucidated, emergence was severely impaired in a few dishes fed with diet 2 (3 dishes) and Tetramin (6 dishes) at 1% and 3% compared to other replicates of the same treatments; these data were removed from the dataset.

**Table 4:** Effect of diet type, diet concentration, larval density and their interactions on the emergence rate

Source	*Df*	*F*	*P*
Diet	3	34.86	< 0.0001**
Concentration	3	42.78	< 0.0001**
Density	3	23.68	< 0.0001**
Diet–concentration	8	22.41	< 0.0001**
Diet–density	9	1.8	0.306
Concentration–density	9	3.99	< 0.0001**
Diet–concentration–density	24	3.14	< 0.0001**

The table describe the results of the logistic analysis (ANOVA) for the effect of diet type (“Diet”), diet concentration (“Concentration”) and larval density (“Density”) on the emergence rate of *An. coluzzii* larvae. *Df* is the degree of freedom, *F* represent the value of the statistical test and *P* is the *P*-value of the test. The symbol “–” indicates the interaction between indicated sources. The symbol “**” indicate a significant effect

**Fig. 3:**
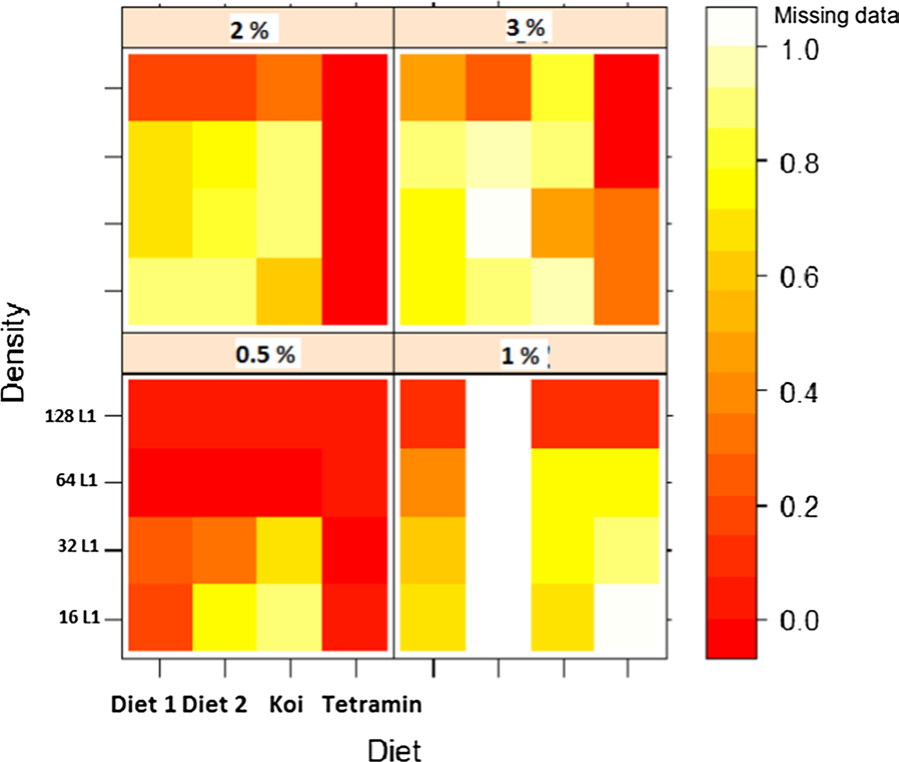
Adult mosquitoes’ emergence rate in relation to diet type, diet concentration and larval density. The figure shows a general overview of the combined effect of diet type, diet concentration and larval density on adult mosquito emergence rate. Each small square represents a combination of a specific number of larvae per Petri dish (16, 32, 64 and 128 first instar larvae) fed with a specific diet (diet 1, diet 2, Koi for the fish food Koi floating blend^®^, and Tetramin for the fish food Tetramin^®^baby), at a specific concentration (0.5, 1, 2 and 3%) represented here by the large square. The adult mosquitoes’ emergence rate is represented by a colour gradient of the small squares, from the bright red (for low emergence rate) to light yellow (for high emergence rate). White indicates missing data

**Fig. 4:**
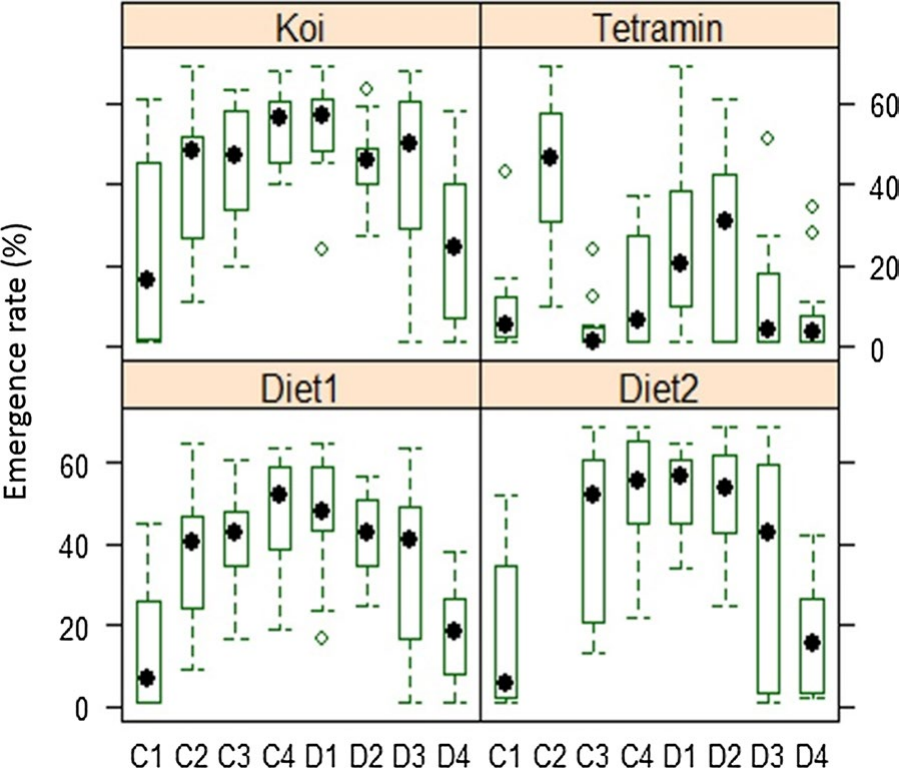
Impact of diet concentration and larval density on adult mosquitoes’ emergence rate (ER) for each type of diet. The figure plots the combined effects of diet type, diet concentration (amount of food provided) and larval density on adult mosquitoes’ emergence rate (number of adults that successfully emerged from an initial number of larvae). The box extends between the 25th and the 75th percentile (across the inter quartile range) and the mean is denoted by a dot

When larvae number was averaged for each diet, the highest emergence rate was seen in treatments fed with Koi (0.575), 2.6 times greater than the emergence rate of treatments fed Tetramin (0.223), considered as the standard in this experiment. The three-way interaction between diet, larval density and diet concentration (Figs. 3, 4) indicated that emergence rate increased with diet concentration and decreased with larval density, suggesting the existence of strong larval competition for food in the conditions tested, especially in treatments with the lower food concentration and the highest larval density.

**Fig. 5:**
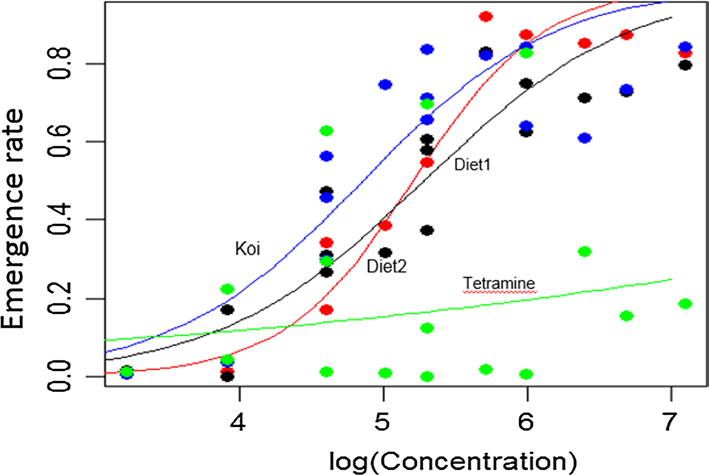
Binary logistic models fitted to adult mosquitoes’ emergence rate (ER) in relation to the amount of food provided. The figure compare the predictive effect of food amount on adult mosquito emergence rate when larvae are fed with the four types of diets: diet 1 (in black), diet 2 (in red) is made of tuna meal (TM), Koi (in blue) is the fish food Koi Floating Blend^®^, and Tetramin (in green) is the fish food Tetramin^®^baby. Concentration represents the amount of food provided per larva per day

A logistic model fitted to the data, where emergence rate is a function of diet concentration, is shown in Fig. 5. Both diet 1 and Koi allowed the emergence of a greater number of adults than diet 2 when fed at the lowest concentration. At a concentration of about log5 the emergence rate of larvae fed diet 2 became greater than those fed diet 1 and surpassed the emergence rate of treatments fed Koi at higher concentrations. Tetramin produced comparable numbers of adults to the other diets at lower diet concentrations but much lower emergence rates at higher diet concentrations, as a result of high larval mortality.

### Wing length

**Fig. 6:**
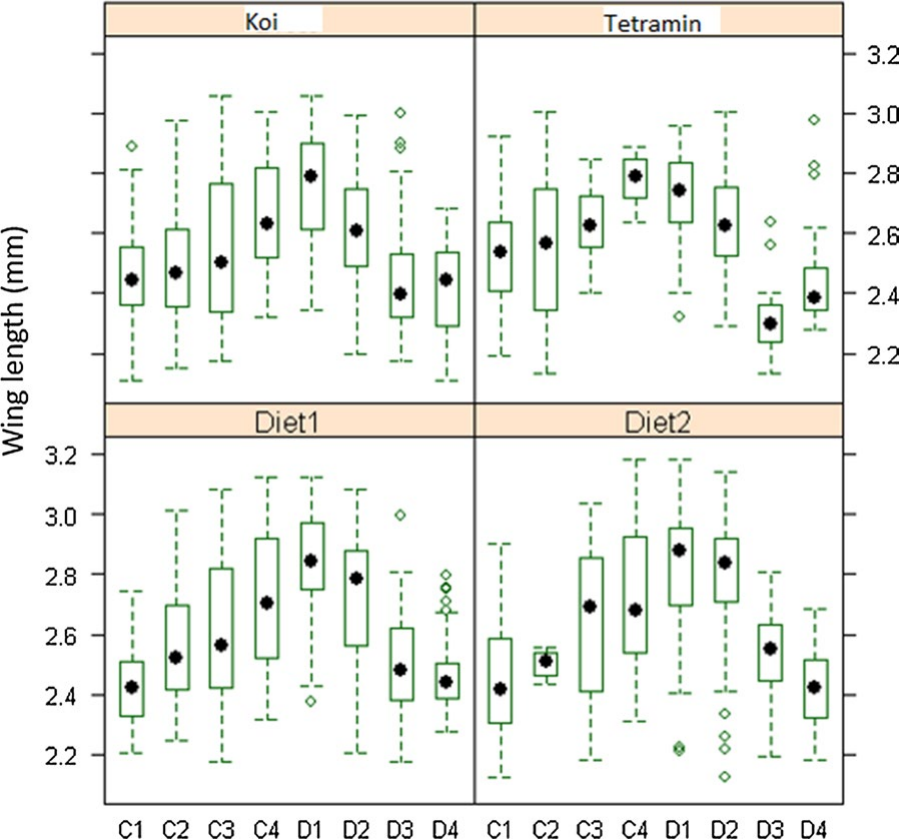
Impact of diet concentration and larval density on wing length (WL) for each type of diet. The figure plots the combined effects of diet type, diet concentration (amount of food provided) and larval density on wing length (as a proxy of mosquito body size). The box extends between the 25th and the 75th percentile (across the inter quartile range) and the mean is denoted by a dot

The variances of wing length were not homogeneous between treatments (*P* < 0.05, Fligner-Killeen test), hence a logarithmic transformation of wing length was performed to proceed with mean wing length. Comparison by an ANOVA test showed strong effects of diet type, larval density and diet concentrations as well as their interactions on wing length (Table 5). The comparison of wing length between treatments fed on different diets is shown in Table 3. Significant differences were found between treatment pairs diet 3–diet 1, diet 3–diet 2 and diet 4–diet 2. Mosquitoes emerging from treatments fed on diet 1 and diet 2 had the longest wings (about 3% longest than mosquitoes from Koi and Tetramin treatments). Overall wing length increased with diet concentration but decreased with larval density (Fig. 6).

**Table 5:** Effect of diet type, diet concentration, larval density and their interactions on wing length

Source	*Df*	*F*	*P*
Diet	3	34.5	< 0.0001**
Concentration	3	143.9	< 0.0001**
Density	3	611.5	< 0.0001**
Diet–concentration	8	5.3	< 0.0001**
Diet–density	9	6.3	< 0.0001**
Concentration–density	9	10.7	< 0.0001**
Diet–concentration–density	24	3.5	< 0.0001**

The table describe the results of the logistic analysis (ANOVA) for the effect of diet type (“Diet”), diet concentration (“Concentration”) and larval density (“Density”) on the wing length of *An. coluzzii* mosquitoes. *Df* is the degree of freedom, *F* represents the value of the statistical test and *P* is the *P*-value of the test. The symbol “–” indicates the interaction between indicated sources. The symbol “**” indicates a significant effect

## Discussion

A good understanding of mosquito larval bio-ecology has proven to be of crucial importance to the effectiveness of vector control measures in the field. This knowledge may also be important to improve larval rearing strategies in mass mosquito production for SIT or other genetic control methods [17]. In this study, the efficacy of different larval diets, were evaluated, as well as their interactions with larval density, in structuring (growth and survival) the *An. coluzzii* larval communities. Data showed significant differences in the development rate, survival to emergence and adult size of mosquitoes fed with each of four different diets, and suggested a negative density-dependent effect of diet availability.

Substantial differences in emergence rate were observed between larvae fed each of the diets. Diets 1, 2 and 3 produced the highest emergence rates when fed at concentrations between 180 and 400 µg/larvae/day, and diet 3 (Koi) produced the highest emergence rate of these three diets in this range. Beyond a concentration of 400 µg/larvae/day, emergence rates of larvae fed with each of the three diets plateaued and then decreased at the highest concentration tested. This phenomenon was also clearly seen with diet 4 (Tetramin). This last diet produced a very poor emergence rate compared to the three other diets, and at high concentrations the diet could not all be consumed by larvae. Consequently, a microfilm was observed at the surface of the water and seemed to kill most of the larvae. Similar observations were previously made by Gilles et al. [21], who showed that 263 µg/larvae/day was the diet availability needed to ensure larval growth, when using the same type of diets for *Anopheles arabiensis* larvae. Adult emergence rate decreased at higher concentrations (increased larval mortality probably because of intoxication due to remaining food).

Increasing larval density also negatively impacted the emergence rate. Two factors may explain this negative impact. First, an increase in larval density, given the same total amount of food, increases competition for food leading to high mortality of larvae that are unable to secure the minimum amount of food needed to secure their growth [16, 28, 29] or because of intra-instar larva cannibalism [30, 31]. Second, an increase in larval density also means reduced space available to an individual larva and when a threshold is reached, an overcrowded larval site can negatively impact development rate [14, 31, 32]. Overcrowded larval breeding sites are rarely seen in nature except when a period of drought shrinks the size of larval habitats. However, because insectaries often lack space, a good balance should be found between the density of larvae, the size of rearing pans and the amount of food provided during rearing to ensure an optimal emergence rate of competitive male adults. In fact, an increase of larval density is known to positively affect cannibalism of *Anopheles gambiae* larvae [31, 32]. This behaviour could be beneficial in nature for it allow development of mosquitoes with a better fitness [30].

Measurements of pre-imaginal developmental time indicated that larvae fed with diet 2 (a mixture of tuna meal, bovine liver powder, squid liver powder, brewer’s yeast and vitamin mix) developed to adult emergence quicker than those fed on the other foods. It is worth noting that though significantly fewer larvae fed on diet 4 (TetraMin^®^Baby) survived to emergence than those fed on diet 3 (Koi Food), no significant difference was observed between them regarding the pre-imaginal developmental time. The greater proportion of larvae developing to emergence (leading to greater congestion of larval environment, so greater larval competition for food) when fed with diet 3 cannot alone explain a longer pre-imaginal developmental time of this diet.

A shorter pre-imaginal development time is highly beneficial in field situations where larvae may experience drought or be exposed to predation. In the insectary, any diet that can shorten pre-imaginal developmental time is welcome as it will reduce the cost (mainly via a reduction in labour costs) and ensure an accelerated production of adults. This is particularly important when considering mass rearing of adult mosquitoes for the SIT or other approaches involving the release of high number of mosquitoes. In agreement with previous studies [28, 33–35], data collected in this study showed that, increasing the amount of available diet accelerates larval development while an increase in larval density leads to longer developmental time, due to intraspecific competition for food and space. It took longer for larvae in low diet concentration treatments to complete their development because larvae exposed to food stress take more time to reach the adult stage [15, 36].

Adult wing length varied depending on the larval diet. Overall larvae fed on diet 2 (mixture of tuna meal, bovine liver powder, squid liver powder, brewer’s yeast and vitamin mix) produced the largest males, suggesting that the nutrient contents of this diet is better than that of the other diets. Several studies have shown that by manipulating the amount of food, one could produce small, intermediate and large size mosquitoes [37]. While the benefits of large females is clear in terms of fecundity [24, 38], it is not very clear yet whether large males have more success in mating than smaller counterparts [39]. Since females malnourished at the larval stage need at least two blood meals before they can oviposit [40], that small malnourished males might need to consume more sugar before they can mate could constitute a reasonable speculation. In that sense, males which are larger at emergence will have an advantage as they may need just one sugar meal before they disperse to search for a mate. However, a large body size may also require the consumption of more nutrients and may reduce flexibility of movement. Some research has shown that intermediate body size correlated to male mating success [39, 41]. In this study both diet concentration and larval density were shown to strongly affect wing length, which increased with diet availability, and decreased with larval density, as has previously been observed [21, 24, 42].

The observed differences in the development of larvae fed on each of the diets suggest that they do not have the same nutritional values. The Koi fish food (diet 3) which has previously been used by the IPCL for mosquito rearing is no longer available. The necessity to find a good substitute to the Koi food has led to a search for a suitable alternative; diets 1 and 2 tested here are the results of those previous studies [22, 23, 43, 44]. It has been demonstrated that a combination of bovine liver powder and tuna meal (present in diets 1 and 2) meet all the essential nutritional requirements of mosquito larvae, including protein, sugar, sterols, vitamins and nucleotides [22]. The same study showed that adding vitamin mix to bovine liver powder and tuna meal significantly improved mosquito growth (survival, development rate and body size). It is thus unsurprising that Tetramin (diet 4), which is less rich than the others in protein and vitamins, produced the poorest developmental performance in larvae to which it was fed.

Production of mosquitoes for SIT requires to face an important challenge: obtaining the highest number of mosquitoes of good quality (good size) at the lowest cost. In Anopheles gender, mosquito size is known to be associated with fecundity [18, 34] and (in less instance) with longevity [34, 45]. It is thus important to ensure at the end of the mass rearing process that mosquitoes produced are of good size. Space (larval density) and food amount available per larvae are important factors that influence mosquito size and thus their quality [17, 22, 34]. Nevertheless, increasing food amount per larvae and space (reduced larval density) will have an important economic cost because of the supplies [46] and a reduced number of mosquitoes produced at a given time. Having a diet that permits an increase of larval density will allow hypothetically increased yield. Similarly, a reduction of pre-imaginal developmental time will reduce the cost via reduction of work and reduction of time for production. Nevertheless, because of complex interactions between diet quality, diet quantity (food amount per tray and per day) and larval density, compromises are always required with the available diet to produce a reasonable number of high quality mosquitoes.

In the context of this study, a general overview of results indicates that best outcomes happen with moderate high values of diet concentration and larval densities, except for mosquitoes fed with Tetramin (diet 4) who performed very badly at high concentrations. So, at the larval density of 64 L1, diet 1 and diet 2 exhibit similar effects on adult emergence rate, pre-imaginal developmental time and wing length with the diet concentrations of 2% and 3%. In fact, at the diet concentration of 2%, all diets gave a similar emergence rate of about 66.28% (54.01–72.50, 95% CIs of the mean), but mosquitoes fed with diet 1 and 2 had an average 4.5% longer wing length than those fed with Koi [2.46 mm (2.42–2.49, 95% CIs of the mean)], and a 15.54% shorter pre-imaginal developmental time [9.20 days (8.97–9.43, 95% CIs of the mean)]. At 3% concentration of diets, the performances of diet 1 and 2 increases with a mean wing length of 2.62 mm (2.59–2.64, 95% CIs of the mean) which was 0.8% longer than mosquitoes fed with Koi, a one day reduced pre-imaginal developmental time [8.21 days (8.09–8.32, 95% CIs of the mean) which was about 10% shorter than those fed with Koi], for an increased adult emergence rate of 85.81% (77.41–94.20, 95% CIs of the mean), comparable (similar) in each diet.

## Conclusion

SIT approaches to control malaria vectors, whether using irradiation or other means of sterilization, have regained interest in recent years. These approaches will require high quality males to be produced *en masse*, sexually competitive and fit enough to withstand adverse natural conditions. A good knowledge of mosquito larval biology is essential to ensure reliable production of suitable males. This study has investigated the impact of different diet types, diet availability and larval density on emergence rate, pre-imaginal developmental time and adult wing length, proxy for adult mosquito size. Diet concentration and larval density were both found to strongly influence mosquito growth rate and adult size. Among the 4 diets, diet 2 (consisting of a suspension of tuna meal, bovine liver powder, squid liver powder, brewer’s yeast and vitamin mix) was considered superior to the others because it produced a good balance between emergence rate, pre-imaginal developmental time and adult size, and was therefore suitable for breeding these mosquitoes. Further studies on the effectiveness of this specific diet under mass rearing conditions are needed, as well as its impact on the successful development of other mosquito species.

## Abbreviations

ANOVA: analysis of variance
df: degree of freedom
FAO: Food and Agriculture Organization
GLM: Generalized Linear Models (GLM)
IAEA: International Atomic Energy Agency
IPCL: Insect Pest Control Laboratory
IRS: indoor residual spraying
IRSS: Institut de Recherche en Sciences de la Santé
ITNs: insecticide-treated nets
L1: first instar larvae
LD: light/darkness ratio
SIT: Sterile Insect Technique
